# Retinal Gene Distribution and Functionality Implicated in Inherited Retinal Degenerations Can Reveal Disease-Relevant Pathways for Pharmacologic Intervention

**DOI:** 10.3390/ph12020074

**Published:** 2019-05-17

**Authors:** Debarshi Mustafi, Amirmohsen Arbabi, Hossein Ameri, Krzysztof Palczewski

**Affiliations:** 1 USC Roski Eye Institute, Department of Ophthalmology, Keck School of Medicine of the University of Southern California, Los Angeles, CA 90033, USA; debarshi.mustafi@case.edu (D.M.); amirmohsenarbabi@gmail.com (A.A.); ameri@med.usc.edu (H.A.); 2 Department of Ophthalmology, Translational Vision Research Center, Gavin Herbert Eye Institute, University of California, Irvine, CA 92697, USA

**Keywords:** retina, retinal gene distribution, retinal gene functionality, inherited retinal degenerations, RNA-Seq, geographic expression, systems pharmacology, drug discovery

## Abstract

The advent of genetic therapies for inherited retinal diseases (IRDs) has spurred the need for precise diagnosis and understanding of pathways for therapeutic targeting. The majority of IRDs that are clinically diagnosed, however, lack an identifiable mutation in established disease-causing loci and thus can be investigated with limited rational drug discovery methods. Transcriptome profiling of the retina can reveal the functional state of the tissue, and geographic profiling can uncover the various clinical phenotypic presentations of IRDs and aid in pharmaceutical intervention. In this investigation, we detail the retinal geographic expression of known retinal disease-causing genes in the primate retina and functional targetable pathways in specific IRDs. Understanding the genetic basis as well as the resulting functional consequences will assist in the discovery of future therapeutic interventions and provide novel insights to medicinal chemists. Herein, we report that, despite the genetic heterogeneity of retinal diseases, potential functional pathways can be elucidated for therapeutic targeting and be used for predictive phenotypic and genotypic modeling of novel IRD presentations.

## 1. Introduction

Inherited retinal degenerations (IRDs) are predominantly monogenic [1], with a prevalence of ~1 in 3000 individuals [2]. There are approximately 300 disease-causing genes that have been discovered to date [3]. The genetic diagnosis of retinal dystrophies has evolved as technology has advanced from Sanger sequencing to current-day next-generation sequencing methodologies. However, diagnosis still remains difficult because of the genetic heterogeneity of retinal dystrophies [4], and this lack of identifiable disease pathways has limited medicinal approaches to drug discovery. Retinitis pigmentosa (RP) is the most common IRD, with over 3000 genetic mutations in approximately 70 genes [5], yet less than 60% of patients have an identifiable mutation in these established disease-causing loci [6], thereby highlighting the need for the discovery of novel loci in monogenic retinal dystrophies [7] for therapeutic targeting. In the case of multifactorial retinal degenerations, even less is known, as multi-genic inheritance patterns and genetic modifiers [8] have confounded molecular diagnosis. Whereas monogenic and multigenic forms of retinal degeneration are distinct, the predominant driving force behind them is a dysfunction at the level of the photoreceptor and the neighboring retinal pigment epithelium (RPE) [9].

The distinct cellular functional features of these photoreceptor cells [10] can give rise to phenotypically varied presentations. Higher order primate retinas are populated with only 5% cone photoreceptors, but their concentration in the macula, most specifically the fovea [11], allows for high-resolution vision. Thus, primary rod photoreceptor dysfunction generally leads to mid-peripheral visual field loss and nyctalopia [12], whereas cone retinopathies more classically lead to central vision loss and a more pronounced decrease in visual acuity [13]. The genes responsible for retinal degeneration are diverse, encompassing all aspects of cellular structure and function. Understanding the mechanistic features has lagged because the genetic dissection of retinal diseases has been limited in the past. Expression profiles of single primate retinal cell types [14] and newly characterized global retinal environments with naturally varying photoreceptor populations [15] have provided insight into the transcriptional framework of the retina in both health and disease states. Whole-exome sequencing, candidate-exome capture, and targeted re-sequencing can only examine the protein-coding component of the genome and are limited for the discovery of novel disease-causing loci. Whole-genome sequencing allows for a more comprehensive analysis and is becoming essential to uncover non-exomic variants in diseases such as Stargardt [16]. RNA-Sequencing (RNA-Seq) allows for the examination of all transcribed sequences, both protein-coding and non-coding [17], while also identifying novel alternative splice isoforms and single-nucleotide polymorphisms. Moreover, the quantitative nature of RNA-Seq allows a reliable detection of those transcripts that are as uniquely expressed as one copy in a cell, provided an appropriate sequencing depth is achieved [18]. Large-scale projects such as ENCODE [19] and GTEx [20] have undertaken the identification of functional genetic elements on both the protein-coding and the non-coding fronts in tissue-specific contexts. Recent large-scale transcriptome-wide association studies have shed light on novel genes associated with age-related macular degeneration [21].

Transcriptome profiling is advantageous in examining the expression level of distinct RNA species to reflect the functional state of the tissue being surveyed. Our results detail the RNA-Seq expression of known retinal disease genes from central and peripheral retinal tissue from the primate *Macaca fascicularis*. The analysis of the trends of geographic expression of individual and overlapping retinal dystrophy genes elucidated the corresponding phenotypic presentation of these diseases by delineating key targetable biological pathways. Furthermore, the examination of newly discovered retinal disease genes illustrated the power of geographic analysis in understanding the phenotypic features of such diseases. Determining the genetic basis of retinal degeneration will be critical in the future to assess a patient’s inclusion in gene therapy trials [22,23] or systems pharmacology therapeutic interventions [24]. With the advent of gene therapy for retinal dystrophies [25] and the geographic limitations of subretinal gene delivery, understanding the pattern of gene expression may be beneficial for medicinal chemists to tailor systemic pharmacologic therapies.

## 2. Materials and Methods

### 2.1. Animal Tissue Isolation

Enucleated macaque (*M. fascicularis*) eyes and retinal tissue were obtained from Ricerca Biosciences (Painesville, OH, USA). All animal procedures and experiments were performed in accordance with U.S. animal protection laws and conformed to the recommendations of both the American Veterinary Medical Association Panel on Euthanasia and the Association for Research in Vision and Ophthalmology.

### 2.2. RNA Isolation

For geographic retinal tissue collection from macaques, each retina was cut into quadrants, and the central region (5.5 mm punch) containing the macula was carefully dissected out with scissors to separate it from peripheral retinal tissue before being placed in RNALater solution for preservation.

### 2.3. Preparation of RNA-Seq Library

Three biological replicates of peripheral retina and four biological replicates of central retina tissue from *M. fasicularis* were isolated for gene expression analysis. Library preparation for Illumina RNA-Seq (Illumina Inc., San Diego, CA, USA) was carried out as previously described [26].

### 2.4. RNA-Seq, Read Mappin, g and Determination of Reads per Kilobase per Million Reads (RPKM)

Libraries of *M. fasicularis* were sequenced using 50-bp read-length paired-end sequencing with the Illumina Genome Analyzer IIx or HiScan SQ. Data were processed and aligned with the University of California–Santa Cruz rhesus (rheMac3) genome assembly and transcript annotations, respectively, using the Genomic Short-Read Nucleotide Alignment Program (GSNAP), manual extraction of uniquely-mapped reads, HTseq for raw read counts of genes, and manual calculation of RPKM [18] statistics by gene. These were further refined manually with RefSeq gene models. Missing homologous genes were identified in the assembly, and reads were mapped to nucleotide sequences from the RefSeq gene models.

The processed and raw FASTQ files for *M. fasicularis* (GSE84929) were previously deposited in the NCBI GEO database and are available for public use.

### 2.5. Pathway Analysis

Pathway analysis and overrepresentation analysis of pathways were carried out using the Protein ANalysis THrough Evolutionary Relationships (PANTHER) classification system and the Reactome pathway database. The overrepresentation analysis was carried out using a Bonferroni correction for multiple testing to report the final *p* value.

### 2.6. Statistical Analyses

The experimental results were analyzed by an independent two-sample t test; *p* values of 0.05 or less were considered statistically significant.

## 3. Results

### 3.1. Geographic Expression Profile of Known Retinal Disease Genes in the Primate Retina

Young, healthy primate *M. fascicularis* were chosen for the study to prevent any confounders of aging or disease in the elucidation of the physiologic genetic landscape of the primate retina. Biological replicates of central and peripheral retinal tissues from *M. fascicularis* sequenced for gene expression analysis revealed 11,059 genes at an expression level ≥ 1 RPKM. Of these, 10,253 genes showed no preferential expression in the center or peripheral retina. As previously shown [15], of the 807 genes that were differentially expressed by at least twofold (*p* value ≤ 0.05), 499 genes were more highly expressed in the central retina, and 308 genes were more highly expressed in the peripheral retina, highlighting the key genetic frameworks instrumental in the function and maintenance of the adult primate retina.

Analysis of *M. fascicularis* retinal tissue revealed the expression (≥1 RPKM) of 186 genes of known human retinal disease-causing genes (RetNet, http://sph.uth.edu/retnet/), whereas the remaining genes were below the detection limit or did not have homologous genes in the rhesus genome assembly or nucleotide sequences from the RefSeq gene models at the time of analysis. Of these 186 genes, 15 were more highly expressed in central retinal isolates, whereas 85 were more highly expressed in the peripheral retina isolates. There were 86 genes that exhibited a pan-retinal expression profile, with no predilection for central or peripheral retina sampling.

**Figure 1:**
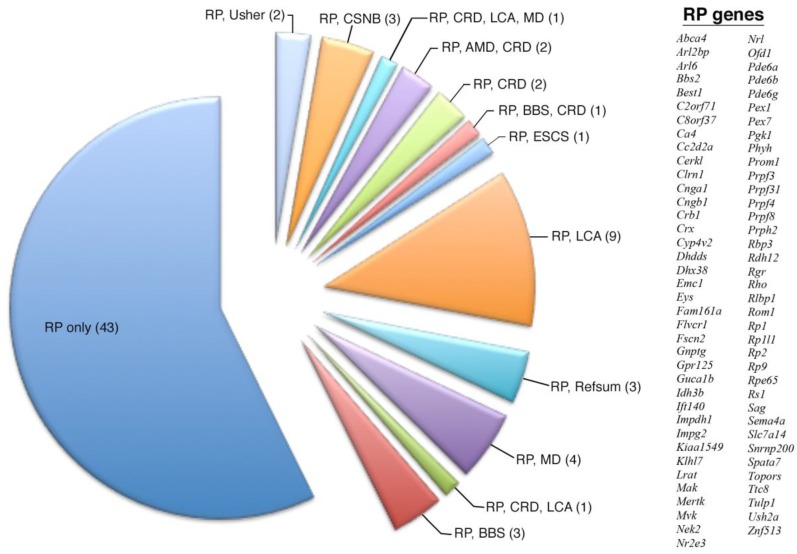
Overlapping expression profile of all 75 retinitis pigmentosa disease-causing genes. The number of total genes is given in parenthesis. Although most of these genes are known to only cause RP, there are many overlapping genetic diseases, with RP and LCA having the most overlapping genes.

The expression profiles of individual and overlapping disease-causing gene loci were further analyzed to reveal specific geographic expression patterns (Table 1). For example, when examining all 75 disease-causing RP genes (Figure 1), there was almost equal predilection for genes displaying pan-retinal versus peripheral expression, but the analysis of overlapping categories such as 9 genes causing both RP and Leber congenital amaurosis (LCA) showed a more biased expression profile in the periphery of the retina (Table 2). The next step in the analysis was to understand if there was any functional or biological basis for these geographic expression profiles of retinal disease genes.

### 3.2. Pathway and Overrepresentation Analysis of RetNet Disease Genes

The 186 RetNet genes expressed in the *M. fascicularis* retinal tissue were categorized for their biological and functional roles with Gene Ontology (AmiGO software) [27] to determine if certain functional categories were overrepresented in our data sets. The use of the PANTHER classification system [28,29] coupled with the Reactome pathway knowledgebase [30] revealed overrepresented pathways in our data set and subsets of disease-causing genes. 

When examining all 186 disease-causing genes that were expressed in our primate retina dataset, the most overrepresented pathway was activation of the phototransduction cascade. It also included distinct pathways for retinoid cycles in cone and rod photoreceptors (Table 3). When examining those disease genes that were more peripherally enriched in the retina, the most overrepresented pathway was again activation of the phototransduction cascade, and, whereas the retinoid cycle pathway in rods was overrepresented, the retinoid cycle in cones was no longer represented. Furthermore, more structural pathways such as BBSome-mediated cargo targeting and degradation of extracellular matrix were enriched in the peripherally expressed disease genes (Table 3). When examining those disease genes that showed pan-expression, the major pathways that were enriched were all related to phototransduction and no longer represented any retinoid cycle pathway genes (Table 3).

The analysis of specific retinal dystrophies such as RP revealed pathway enrichment related to gene expression location and disease overlap/heterogeneity. In the examination of all 75 RP disease-causing genes, we found an enrichment of the phototransduction (*Cnga1*, *Cngb3*, *Guca1b*, *Pde6a*, *Pde6b*, *Rho*, *Sag*) and rod retinoid pathway genes (*Abca4*, *Lrat*, *Rdh12*, *Rbp3*, *Rlbp1*, *Rpe65*) (Table 4). When the subgroup of the 33 peripherally enriched and 38 pan-expressed RP genes was examined, although both displayed enrichment of phototransduction and rod retinoid pathway genes, the pan-expressed genes were also enriched for cargo trafficking genes. Further analysis with the PANTHER module revealed that the pan-expressed but not the peripherally expressed RP genes showed enrichment in RNA-splicing pathway genes (Table 4).

When examining the genetic overlap of disease in RP, we found that in those 43 genes that are only known to cause RP, there was enrichment not only of genes in the phototransduction and retinoid pathways, but also of genes involved in mRNA splicing, such as *Prpf3*, *Prpf4*, *Prpf8*, and *Prpf31* (Table 4). Out of those 43 peripheral and pan-expressed genes, we found that the main difference was the enrichment of cargo trafficking genes in the pan-expressed genes. When examining the nine overlapping genes known to cause both LCA and RP (*Crb1*, *Cr*, *Ift140*, *Impdh1*, *Lrat*, *Prph2*, *Rdh12*, *Rpe65*, *Rs1*, *Spata7*, *Tulp1*), we found enrichment in the retinoid cycle in rods as the predominantly overrepresented pathway (Table 4), similar to what was found when examining all 22 genes known to cause LCA.

Further analysis of genetic variants of RP implicated in autosomal dominant (ADRP) versus autosomal recessive (ARRP) [31] forms of the disease revealed specific geographic and pathway enrichment profiles. Of the 20 genes examined to cause ADRP, the majority displayed no geographic predilection in the expression profile, with the remaining seven exhibiting more peripheral expression. In comparison with the 32 genes examined to cause ARRP, the majority, encompassing 20 genes, exhibited enrichment in expression in the peripheral retina, whereas only 2 genes showed central retinal enrichment, and the remaining 10 genes displayed no geographic predilection. The analysis of the pathway enrichment revealed that the pathways implicated in ADRP showed enrichment in RNA-splicing pathway genes such as *Prpf3*, *Prfp8*, *Prpf31*, but this pathway was not enriched in ARRP disease-causing genes (Table 5). Further elucidation of the functionally relevant pathways revealed retinoid cycle disease events and retinoid cycle in cones were enriched exclusively in ARRP (Table 5). 

## 4. Discussion

Retinal degenerations can be broadly categorized as primarily affecting rod or cone photoreceptors or as involving both and may be further subdivided into progressive or stationary disorders. Understanding the mechanistic pathways of these distinct diseases is essential to ameliorate these blinding conditions by allowing for rational drug design approaches. Our dataset revealed the retinal geographic expression of known retinal disease-causing genes in the primate retina. Many genes displayed no predilection for either the periphery or the center of the retina, exhibiting a pan-retinal expression pattern. Of the genes that were more peripherally enriched, the majority was expectantly implicated in diseases primarily affecting rod photoreceptors, including retinitis pigmentosa, rod-cone dystrophies, and ciliopathies such as Bardet–Biedl syndrome and Usher syndrome (Table 1). More interestingly, when we examined our data for genetic loci that had overlapping disease indications, we found that there was geographic enrichment for certain disease associations. As outlined in Table 2, when all RP-causing genes were examined, there was an equal split between peripherally enriched genes and pan-retinal genes. However, in those genes implicated in RP and LCA, one could appreciate an enrichment in the peripheral expression of that subset of genes.

Often, the genes implicated in retinal degeneration can have a heterogenic phenotypic profile. One may expect a disease instigating retinitis pigmentosa, primarily a rod degeneration affecting peripheral vision initially, would not overlap with macular dystrophy (MD), which, in contrast, afflicts the central cone photoreceptors and affects central vision. However, there are four genes expressed in our dataset that are implicated in both RP and MD. Of these four genes, our data indicated that three genes exhibited a pan-expression profile, with one showing a geographic enrichment in the retinal periphery. Thus, in retinal disease, it is not necessarily the predilection of geographic gene expression, but rather disease-specific mutations that may drive distinct phenotypes [32]. Thus, to better understand the functional aspect of retinal diseases and how they relate to their geographic expression profile in the retina, we carried out pathway analysis to comprehend the functional dynamics that may drive these distinct phenotypic patterns.

**Figure 2:**
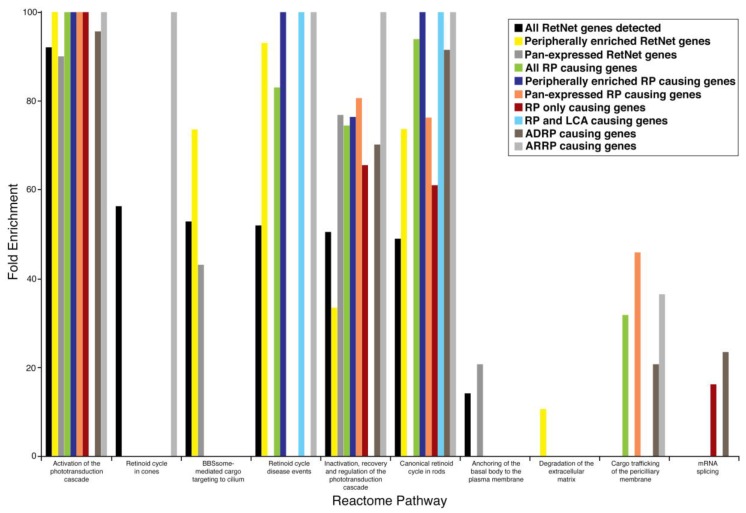
Enrichment profile of reactome pathways in different subsets of retinal disease-causing genes and their geographical expression profile in the retina. Abbreviations: ADRP (autosomal dominant retinitis pigmentosa), ARRP (autosomal recessive retinitis pigmentosa).

Pathway analysis of the expressed retinal disease genes in the primate retina elucidated specific functional pathways that were implicated in a geographic context. Compared to all expressed disease genes in the retina, those that were more peripherally expressed displayed enrichment for rod retinoid cycle genes and genes involved in the degradation of the extracellular matrix (*Adam9*, *Adamts18*, *Capn5*, *Col2a1*, *Col9a1*, *Col11a1*), a common remodeling mechanism related to the phagocytosis and daily renewal burden of rod photoreceptors implicated in disease [26]. Interestingly, all three collagen genes (*Col2a1*, *Col9a1*, *Col11a1*) related to extracellular matrix degradation and enriched in expression in the peripheral retina are known to cause the Stickler Syndrome. In contrast, when examining those disease genes that exhibited pan-expression in the retina, there was a more limited implication of the canonical rod retinoid pathway and a larger implication of pathways related to the phototransduction cascade and of structural pathways of cilium and plasma membrane maintenance (*Ahi1*, *Arl6*, *Cep164*, *Ift140*, *Sdccag8*, *Rpgrip1l*). Again, the most interesting findings arose from the analysis of genetic loci that had overlapping disease indications (Figure 2).

The examination of the most robust disease, RP, revealed distinct functional categories that related not only to geographic expression but also to gene loci with overlapping disease associations. It is noteworthy to mention that when all pan-expressed disease genes were examined, the canonical retinoid cycle in rods was not enriched functionally; however, in RP, whether we examined those genes that were peripherally expressed or those that were pan-expressed, both geographic patterns of this disease exhibited enrichment of the pathway for the canonical retinoid cycle in rods. This highlights the importance of this pathway in retinitis pigmentosa, as it encompasses all patterns of this disease, and its immense potential in future pharmacologic intervention. Targeting this overarching pathway with novel therapeutics can thus ameliorate many clinically diverse presentations of RP. The analysis of mRNA splicing in those genes that are only involved in RP, in the subset of all RP genes that are pan-expressed, as well as in those ADRP disease-causing genes, indicated a functionally distinct pathway that may be implicated in this disease and that may localize both geographically and genetically in certain populations. When we examined newer genes that had more recently been added to the RetNet database, we found that one such new gene, the *AhR* (aryl hydrocarbon receptor) gene, was indicated to cause RP as a consequence of aberrant mRNA splicing. Studies using *Ahr* knockout mice have demonstrated late-onset retinal degeneration [33].

Functional pathway analysis of gene loci with overlapping disease associations also highlighted disease-specific pathways. When examining the overlapping RP and LCA genes, not only were they more peripherally expressed, but there was also a clear enrichment of rod retinoid cycle functionality for these disease genes. One such gene, *RPE65* (retinal pigment epithelium-specific 65 kDa protein, also known as retinoid isomerase), is the subject of the first successful FDA-approved gene therapy trial [23]. Furthermore, understanding that the rod retinoid pathway is disrupted in these retinal diseases has also led to pharmacologic treatment options [34,35] shown in clinical trials to benefit not only patients with RP and LCA that possess a defective *Rpe65* gene, but also patients that possess a defective *Lrat* (Lecithin retinol acyltransferase) gene [24]. This highlights that therapeutic targeting of underlying pathways can be used to treat diseases with several genetic causes, despite the heterogeneity of disease manifestations. Moreover, the rational targeting of disease pathways elucidated from this study can potentially treat multiple overlapping retinal dystrophies.

In search of causative genes in patients with clinical features of RP, sequencing of families led to the discovery of the disease genes *Adipor1* (Adiponectin receptor 1) and *Reep6* (Receptor Accessory Protein 6) that lead to syndromic RP [36] and autosomal recessive RP [37], respectively. Interestingly, the examination of our RNA-Seq dataset examining the expression levels of retinal genes across species with several distinct photoreceptor populations had previously implicated *Adipor1* and *Reep6* as enriched in the rod photoreceptor clusters by whole-genome network analysis [15] and predicative of a retinitis pigmentosa-like phenotype, as has since been confirmed. Thus, large-scale transcriptome sequencing and network analysis have the power to predict possible phenotypic presentations of aberrant genetic changes in the population, which can lead to potentially novel pathways for therapeutic intervention [21].

Genetic approaches have the potential to shed light on more than single gene associations; they have the potential to reveal overarching pathways of disease that can be targeted pharmacologically in addition to genetic modifications. Single genes and their resulting functional impacts on the retina can be targeted with gene therapy. However, this is an invasive technique and as of now can be reliably transduced only in a limited geographic setting in the subretinal space of injection. RNA differences either at the expression or at the nucleotide level seemingly drive the observed phenotypic diversity of retinal diseases. Understanding the genetic basis as well as the resulting functional consequences will assist in therapeutic intervention in the future. Whole-genome approaches reveal novel rare variants in coding regions as well as in noncoding regions [38] implicated in retinal homeostasis from rodents to primates [39] and, specifically, in RP pathogenesis [40]. Thus, analytic approaches must expand from single-gene comparisons to system-level approaches to reveal the functional relevance of novel genes as drivers of this phenotypic patterning. 

Systems pharmacologic approaches have already shown much success in targeting retinal diseases such as Stargardt disease [41,42] and came from the elucidation not only of the disease -specific genes, but also of the underlying retinoid cycle pathway and retinal degeneration driven by G-protein coupled receptors. Our study demonstrates that the genetic heterogeneity of retinal diseases could be better understood to reveal functional pathways that can potentially be targeted in overlapping forms of retinal disease. Knowledge gained from this study lays the groundwork for the future predictive phenotypic modeling of new disease-causing genes in the retina and for guided therapeutic intervention.

A recent demonstration of a genetically tiered testing strategy can improve our genetic diagnosis of retinal disease and facilitate treatment [43]. Moreover, the use of weighted whole-genome approaches can reveal candidate eye disease genes [44]. The use of patient-derived induced pluripotent stems cells [45] and the retinal organoid technology [46,47] offer the promise to allow experimentation of disease-associated mutations in a genetically controlled context. Studying specific disease-associated variants in an otherwise identical isogenic context has incredible potential to detect even subtle molecular or cellular phenotypes masked by variability in the genetic background. These evolving cell-based approaches will facilitate unbiased high-throughput compound- or genome-scale genetic screens, which will allow one to focus on screens that reverse complex disease-associated phenotypes, even when limited knowledge exists regarding the underlying disease mechanism. Together, these genetic tools provide incredible promise for medicinal chemists to treat retinal blinding diseases in the future.

## Figures and Tables

**Table 1:** Geographic expression profiles of retinal disease genes.

Disease	Central	Peripheral	Pan-Retinal	Total
AMD	1	7	3	11
BBS	1	11	8	20
CSNB	1	5	8	14
LCA	0	11	11	22
MD	2	5	4	11
Optic Atrophy	1	1	3	5
Refsum Disease	0	2	1	3
RP	4	33	38	75
Usher Syndrome	1	6	3	10
Stickler Syndrome	0	3	0	3
Joubert Syndrome	0	3	4	7
Senior–Loken Syndrome	0	3	2	5
Other	2	9	13	24

Abbreviations: AMD (age-related macular degeneration), BBS (Bardt–Biedl Syndrome), CSNB (congenital stationary night blindness), LCA (Leber congenital amaurosis), MD (macular dystrophy), RP (retinitis pigmentosa), Other (all remaining diseases with less than three genetic loci).

**Table 2:** Geographic expression profile of all retinitis pigmentosa disease-causing genes.

Disease	Central	Peripheral	Pan-Retinal	Total
RP	4	33	38	75
RP only	4	18	21	43
RP, BBS	0	1	2	3
RP, CRD, CA	0	0	1	1
RP, MD	0	1	3	4
RP, Refsum	0	2	1	3
RP, LCA	0	6	3	9
RP, ESCS	0	1	0	1
RP, BBS, CRD	0	0	1	1
RP, CRD	0	1	1	2
RP, AMD, CRD	0	2	0	2
RP, CRD, LCA, MD	0	0	1	1
RP, CSNB	0	1	2	3
RP, Usher	0	2	0	2

Abbreviations: CRD: cone-rod dystrophy, ESCS: enhanced S-Cone syndrome, CSNB: Congenital stationary night blindness.

**Table 3:** Pathway and overrepresentation analysis of all genes (186) and of the peripherally enriched versus the pan-expressed genes.

Reactome Pathway	Fold Enrichment	*p* Value
**All expressed RetNet genes (186)**		
Activation of phototransduction cascade	92.07	<0.00001
The retinoid cycle in cones	56.26	0.04240
BBSome-mediated cargo targeting to cilium	52.82	<0.00001
Retinoid cycle disease events	51.93	<0.00001
Inactivation, recovery, and regulation of the phototransduction cascade	50.44	<0.00001
The canonical retinoid cycle in rods	48.92	<0.00001
Anchoring of the basal body to the plasma membrane	14.07	<0.00001
**Peripherally enriched RetNet genes (85)**		
Activation of phototransduction cascade	>100	<0.00001
Retinoid cycle disease events	93.02	<0.00001
BBSome-mediated cargo targeting to cilium	73.61	<0.00001
The canonical retinoid cycle in rods	73.61	<0.00001
Inactivation, recovery, and regulation of the phototransduction cascade	33.36	0.01300
Degradation of the extracellular matrix	10.59	0.04350
**Pan-expressed RetNet genes (86)**		
Activation of phototransduction cascade	90.02	0.00026
Inactivation, recovery, and regulation of the phototransduction cascade	76.83	<0.00001
BBSome-mediated cargo targeting to cilium	43.05	0.00478
Anchoring of the basal body to the plasma membrane	20.63	0.00001

**Table 4:** Pathway and overrepresentation analysis of RP-causing genes.

Reactome Pathway	Fold Enrichment	*p* Value
**All RP causing genes (75)**		
Activation of phototransduction cascade	>100	<0.00001
The canonical retinoid cycle in rods	93.93	<0.00001
Retinoid cycle disease events	83.01	0.00036
Inactivation, recovery, and regulation of the phototransduction cascade	74.42	<0.00001
Cargo trafficking of the periciliary membrane	31.74	0.00008
**Peripherally enriched RP genes (33)**		
Activation of phototransduction cascade	>100	<0.00001
Retinoid cycle disease events	>100	0.00002
The canonical retinoid cycle in rods	>100	<0.00001
Inactivation, recovery, and regulation of the phototransduction cascade	76.38	0.00046
**Pan-expressed RP genes (38)**		
Activation of phototransduction cascade	>100	0.00179
Inactivation, recovery, and regulation of the phototransduction cascade	80.62	0.00036
The canonical retinoid cycle in rods	76.24	0.01610
Cargo trafficking of the periciliary membrane	45.84	0.00339
**Genes causing only RP (43)**		
Activation of phototransduction cascade	>100	0.00002
Inactivation, recovery, and regulation of the phototransduction cascade	64.50	0.00091
The canonical retinoid cycle in rods	60.99	0.03180
mRNA splicing	16.12	0.00350
**Overlapping genes causing RP and LCA (9)**		
Retinoid cycle disease events	>100	0.02430
The canonical retinoid cycle in rods	>100	0.00019

**Table 5:** Pathway and overrepresentation analysis of genetic variants of RP-causing genes.

Reactome Pathway	Fold Enrichment in ADRP (*p* Value)	Fold Enrichment in ARRP (*p* Value)
mRNA Splicing	23.38 (0.00002)	-
Activation of phototransduction cascade	95.65 (0.0113)	>100 (<0.00001)
Retinoid cycle disease events	-	>100 (<0.00001)
The canonical retinoid cycle in rods	91.49 (0.00025)	>100 (<0.00001)
The retinoid cycle in cones	-	>100 (0.00009)
Inactivation, recovery, and regulation of the phototransduction cascade	70.14 (0.00042)	>100 (<0.00001)
Cargo trafficking to the periciliary membrane	20.63 (0.0483)	36.4 (0.00009)

## Abbreviations

AhR, aryl hydrocarbon receptor; AMD, age-related macular degeneration; BBS, Bardt–Biedl Syndrome; CSNB, congenital stationary night blindness; LCA, Leber congenital amaurosis; MD, macular dystrophy; RP, retinitis pigmentosa.
